# Supplementary material for: Comparative performance of different methods for circulating tumor cell enrichment in metastatic breast cancer patients
Source: PLoS One. 2020 Aug 13;15(8):e0237308. doi: 10.1371/journal.pone.0237308 (PMC7425969; doi:10.1371/journal.pone.0237308)
Supplement: S1 Table — (PDF) [file pone.0237308.s001.pdf]

Summary of published studies of comparative analysis with RosetteSep™ and ScreenCell® Cyto kits

Paper(ref)	CTC isolation technique	Validation Spike in	Recovery (%)	Tumor type	Clincical validation	Method for CTC enumeration	Comments
He at al, 2009 (22)	RosetteSep	OGROV	62	Metastatic Ovarian cancer	18/20 (90%)	Flow cytometry	Compares CTC isolation methods based on altering cells and separating by biophysical properties. Methods include Ficoll Paque, A23187 + Ficoll-Paque, RosetteSep-Ficoll, Ammonium chloride lysis, histopaque, OncoQuick and Leucosep tube with Ficoll
		LNCaP	59	Metastatic Prostate cancer	10/13 (77%)		
Kulashinge et al, 2016 (23)	RosetteSep	Fadu Cells (EpCAM + cells)	70	Advanced Head and Neck cancer	16/25 (64%)	ICC	Both techniques were better than CellSearch in detection CTCs. No head-to-comparison of RS vs SC in patient samples was perfromed.
	ScreenCell		60		13/28 (46.4%)		
	CellSearch		95		8/43 (18.6%)		
Maertens et al, 2017 (24)	EpCAM positive selection	CAL-54; CAKI-1; CAKI-2; A-498	23	Clear Cell Renal Carcinoma	No clinical data	Flow cytomertry coupled to fluorescence microscopy	Used different cell lines with variable EpCAM expression to show CTCs with variable EpCAM expression are missed with antigen surface capture methods. Median cell recovery of all cell lines shown.
	Density based +CD45 negative selection		55				
	RosetteSep		30				
	Parsotix (size and deformability)		66				
