# Supplementary material for: Comparative performance of different methods for circulating tumor cell enrichment in metastatic breast cancer patients
Source: PLoS One. 2020 Aug 13;15(8):e0237308. doi: 10.1371/journal.pone.0237308 (PMC7425969; doi:10.1371/journal.pone.0237308)
Supplement: S2 Table — (PDF) [file pone.0237308.s002.pdf]

Trial runs with combined Dynabeads of CD45 and EpCAM, and combined EasySep nanoparticles of CD45

	Dynabeads	EasySep	
**Trial #**	**1000**	**1000**	**0**
	# of cells	# of cells	# of cells
1	54	36	0
2	131	0	0
3	218	24	0
4	22		0
5	38		

Trial runs with RosetteSep Human Circulating Epithelial Tumor Cell Enrichment Cocktail

**Trial #**	**1000**	**100**	**10**	**0**	**1000 + EpCAM**
	# of cells	# of cells	# of cells	# of cells	# of cells
1	512			0	114
2	435				169
3	385	67			34
4	333	21		0	40
5	116	34	2		
6	164	33	7		
7	436	19	2	0	
		44	5	0	

Trial runs of ScreenCell

Trial #	100 cells	0 cells
1	42	0
2	73	0
3	50	0

and EpCAM, respectively
