# Supplementary material for: Comparative performance of different methods for circulating tumor cell enrichment in metastatic breast cancer patients
Source: PLoS One. 2020 Aug 13;15(8):e0237308. doi: 10.1371/journal.pone.0237308 (PMC7425969; doi:10.1371/journal.pone.0237308)
Supplement: S3 Table — (PDF) [file pone.0237308.s003.pdf]

CTC isolation from patient plasma samples

	subject	SC CTC	RS CTC	Vol RS (ml)	CTCs	CTCs/ml	SC
1	CTC002-1	n/a	2	5.3	Y	0.38	
2	CTC002-2	n/a	2	5.5	Y	0.36	
3	CTC002-3	n/a	0	5.3	N	0	
4	CTC002-4	n/a	2	5	Y	0.40	
5	CTC002-5	n/a	0	5	N	0	
6	CTC004-1	n/a	?	5	N	Inconclusive	
7	CTC004-2	n/a	?	5	N	Inconclusive	
8	CTC009	n/a	1	5.3	N	0.19	
9	CTC011	n/a	2	5	Y	0.40	
10	CTC012	n/a	11	5	Y	2.20	
11	CTC014	n/a	16	5	Y	3.20	
12	CTC014-2	n/a	25	5	Y	5	
13	CTC017	n/a	0	5	N	0	
14	CTC018	n/a	0	5	N	0	
15	CTC019	n/a	4	5	Y	0.80	
16	CTC020	n/a	0	5	N	0	
17	CTC021	n/a	0	5	N	0	
18	CTC022	n/a	0	5	N	0	
19	CTC023-1	n/a	0	5	N	0	
20	CTC023-2	n/a	2	4.5	Y	0.44	
21	CTC023-3	0	1	4.5	Y	0.22	0
22	CTC024-1	n/a	0	5	N	0	
23	CTC024-2	2	2	4.25	Y	0.47	0.67
24	CTC025	n/a	0	5	N	0	
25	CTC028	n/a	1	5.5	Y	0.18	
26	CTC029	n/a	3	6.25	Y	0.48	
27	CTC030	0	0	5.5	N	0	0
28	CTC031	0	0	6.5	N	0	0
29	CTC032	2	2	4.5	Y	0.44	0.67
30	CTC036	4	n/a	3	Y		1.33
31	CTC037	0	1	4.15	Y	0.24	
32	CTC038	118	n/a	3	Y		39.33

ScreenCell (SC) only (green shading)

ScreenCell (SC) and RosetteSep (RS) (yellow shading)

n/a not available
