# Supplementary material for: Comparative performance of different methods for circulating tumor cell enrichment in metastatic breast cancer patients
Source: PLoS One. 2020 Aug 13;15(8):e0237308. doi: 10.1371/journal.pone.0237308 (PMC7425969; doi:10.1371/journal.pone.0237308)
Supplement: Attachment — Submitted filename: Response to Reviewers.docx [file pone.0237308.s004.docx]

**Response to reviewers**

November 20, 2019

Dr. Sophia N. Karagiannis

Academic Editor

PLOS ONE

Dear Dr. Karagiannis

**Re: Revisions to PONE-D-19-23597**

We thank you and the reviewers for the constructive feedback. This letter addresses your concerns and those raised by the reviewers to our manuscript entitled “Comparative performance of different methods for circulating tumor cell enrichment in metastatic breast cancer patients”. We have also modified the manuscript and declarations in order to conform to PLOS ONE publication criteria as requested. We have also included a marked-up (with track changes) and clean (without track changes) document files as requested by PLoS. A detailed response to each point is appended to this letter (our response is italicized). We would be grateful if you would consider the revised manuscript for publication in PLOS ONE.

On behalf of the Study authors,

Sincerely,

Arik Drucker MD FRCPC

Journal Requirements

1. Please ensure that your manuscript meets PLOS ONE's style requirements, including those for file naming. The PLOS ONE style templates can be found at…

*Style and Author affiliation style templates were followed and several changes were made to the manuscript. See “Revised Manuscript with Tracked Changes” for full details.*

2. At this time, we ask that you please add scale bars to the microscopy images provided in the manuscript and add the scale represented in the appropriate figure legend. Thank you for your attention to this request.

*Scale bars were added to all images.*

3. In your Methods section, please provide additional information in the your Methods section about the dates (month/year) of enrolment of the breast cancer patients used in this study. Additionally, please provide additional details regarding the breast cancer patients consent.

*The requested information has been added to the Methods section, see lines 175-184.*

4. We suggest you thoroughly copyedit your abstract for language usage, spelling, and grammar. If you do not know anyone who can help you do this, you may wish to consider employing a professional scientific editing service.

*The manuscript was thoroughly proofread by the authors.*

5. We note that you have indicated that data from this study are available upon request. PLOS only allows data to be available upon request if there are legal or ethical restrictions on sharing data publicly. For information on unacceptable data access restrictions, please see….

*We have added a table containing the data from this study in the Supporting Information*

6. We note that you have included the phrase “data not shown” in your manuscript. Unfortunately, this does not meet our data sharing requirements. PLOS does not permit references to inaccessible data. We require that authors provide all relevant data within the paper, Supporting Information files, or in an acceptable, public repository. Please add a citation to support this phrase or upload the data that corresponds with these findings to a stable repository (such as Figshare or Dryad) and provide and URLs, DOIs, or accession numbers that may be used to access these data. Or, if the data are not a core part of the research being presented in your study, we ask that you remove the phrase that refers to these data.

*We have added a table containing the data from this study in the Supporting Information*

7. Please remove any funding-related text from the manuscript and let us know how you would like to update your Funding Statement. Currently, your Funding Statement reads as follows:

*Changes completed.*

8. We note that you received funding from a commercial source: Pfizer Oncology IIR

Please provide an amended Competing Interests Statement that explicitly states this commercial funder, along with any other [relevant declarations](http://www.plosone.org/static/competing.action) relating to employment, consultancy, patents, products in development, marketed products, etc.

Within this Competing Interests Statement, please confirm that this does not alter your adherence to all PLOS ONE policies on sharing data and materials by including the following statement: "This does not alter our adherence to PLOS ONE policies on sharing data and materials.” (as detailed online in our guide for authors <http://journals.plos.org/plosone/s/competing-interests>).  If there are restrictions on sharing of data and/or materials, please state these. Please note that we cannot proceed with consideration of your article until this information has been declared.

Please include your amended Competing Interests Statement within your cover letter. We will change the online submission form on your behalf.

*Changes completed.*

Reviewer #1

Reviewer #1: The manuscript(MS#PONE-D-19-23597) mainly compared the potential of four methods in the CTCs identification in order to find a simpler and faster method. The methods provided a potential for clinical practice. Actually, several methods have been reported to determine CTCs for cancer prognosis and diagnosis. Therefore, what is the novelty of the developed method? Some other issues should be addressed.

*The novelty of this manuscript lies in the head-to-head comparison of various CTC isolation techniques using both spiking of cultured cells into healthy donor blood and the analysis of paired clinical samples using the most sensitive techniques (Rosette Sep and Screencell). We have modified the manuscript to better explain the utility of the findings relative to published methods.*

1. Two cell lines (MDA-MB-231 and MCF-7) were selected in this study, but why was only MDA-MB-231 used in “Spiking experiments with healthy controls”?

*MDA-MB-231 cells are derived from a patient with triple negative disease and this sub-type of breast cancer produces CTC are generally smaller and more difficult to detect compared to MCF-7 cells. To avoid confusion, we have deleted the MCF-7 cell lines from the methods section.*

2. How to ensure that the cells isolated are the target tumor cells?

*We used accepted criteria for designation of cells as CTCs. This information is contained in the lines 222-225 of the manuscript. “Cells with pan-CK/CK-7^+^/ CD45^-^ and an intact nucleus with DAPI staining were defined as a CTC and counted manually. In addition to the staining criteria, cells were also evaluated based on the cell size (> 5 µm) and cytomorphological appearance such as enlarged nuclear size with high nuclear to cytoplasmic ratio, especially when CK staining was variable.”*

3. Please provided the detailed figure legends. All the figures are not unambiguous, please make all the figures

*All figures and figure legends were improved.*

4. Please explain why there are different experiment times in different tests, as shown in Figure 2B.

*The amount of blood obtained was variable in volume and we also had a 4 hour time limit between blood collection & processing. These restrictions caused us to conduct experiments with a flexible time frame.*

5. In Table 2, the total time of ScreenCell should be ‘4h 15min’, but not ‘4h 7min, as well as Line 259.

*Error has been corrected.*

6. Please make a short conclusion for this research.

*The following short conclusion was added (lines 444-454).*

*The RosetteSep™ and ScreenCell® systems are able to isolate CTCs and CTC clusters from metastatic breast cancer patients. These techniques also provide adequate sensitivity at levels as low as 2 CTCs/mL, which is not the case for the Dynabead® and EasySep^TM^ techniques. The independence of EpCAM expression and simple operation using standard laboratory equipment render the RosetteSep™ and ScreenCell® techniques amenable to routine analysis in clinical settings. The results presented her warrant further investigation of in larger, prospective studies in order to further explore the utility of CTC analysis as an actionable assay in clinical settings.*

Reviewer #2: Drucker et al evaluated four techniques for isolation and analysis of CTCs and found that these systems had comparable performance when tested using high concentrations of cultured cell spike-in models. Two techniques fared better at lower concentrations and were tested using clinical blood samples from patients with metastatic breast cancer.

The experimental design is not clear, and the results are difficult to follow. The goal of comparing different CTC techniques is not novel. The authors could have designed a novel approach to head-to-head testing of different platforms, but this was not the case.

*The manuscript has been modified to clarify the experimental design, specifically a more detailed study design was is now included in Figure 1. Recent literature is presented in the introduction in order to place this study in the context of similar published studies and to demonstrate the novelty of the results.*

Introduction:

Reference 6. There are more recent reviews on CTC isolation. Please update your literature citations.

Refence 10. Please provide an appropriate citation regarding low expression of EPCAM in certain tumor types, e.g., triple negative and NSCLC.

*Three additional references have been added as requested.*

Line 62-64. A non-sequitur. It is not clear what that point of these sentences are. Are these the rationale why you did not benchmark your experiments with an FDA-cleared test and compared correlation with clinical outcomes?

*This section briefly describes the challenges of current CTC isolation techniques, especially the commonly used CellSearch positive selection technique in order to highlight the need for further research. It has been modified to improve clarity.*

This head-to-head comparison study is not unique, please explain how your study is unique. Please also provide a supplementary table that summarizes the results from other related comparative studies.

*A supplementary table is now provided that summarizes other comparative studies. These studies have not compared the ScreenCell and RosetteSep techniques head-to-head using clinical samples. We trust that this additional information more clearly conveys the novelty of this study.*

Materials and Methods:

The number of spike-in cells is too high (10-100 fold higher) and does not reflect the clinical setting with very limited number of cells detected (approximately <1CTC/mL in metastatic breast cancer).

*The reviewer is correct that high spike in levels are not reflective of the clinical setting. However, low spike in levels resulted in high variance. Therefore, high spike in levels were only used to minimize the variance and enable quantitative comparison of the various techniques. This is a common approach in many published studies. The level of 10 cells in 5 mL is similar to levels observed in our patient cohort.*

The experimental design is unclear. Please add a Figure 1b to illustrate study schema or the workflow of experiments. The current figure only describes the techniques.

*Agreed. Additional details were added to Figure 1 as suggested in order to improve the clarity of the experimental design.*

Results:

Genomic testing of cells isolated in MBC patients were not performed so it is unclear whether cells detected by these methods are truly of malignant origin or may be just false positives. For examples, FISH test of CTCs from HER2+ patients could have been performed to address this question.

*We agree that Her2 FISH testing would be useful for this subset of patients, however, additional markers would be needed for other subtypes increasing the complexity of the analysis. We used nucleus and pan cytokeratin staining. These are well accepted criteria for designation of cells as CTCs. We also included both positive and negative controls in each experiment. This information is contained in the lines 222-225 of the manuscript. “Cells with pan-CK/CK-7^+^/ CD45^-^ and an intact nucleus with DAPI staining were defined as a CTC and counted manually. In addition to the staining criteria, cells were also evaluated based on the cell size (> 5 µm) and cytomorphological appearance such as enlarged nuclear size with high nuclear to cytoplasmic ratio, especially when CK staining was variable.”*

Please provide a supplementary table (CTC yields) that shows all the results of the experiments. This should be made available.

*Supplementary Table 2 has been added*

The X's before number of spiked cells in Figure 2B are R artifacts. Please clean up.

*Error has been corrected.*

The negative x-axis range in Figure 5B is confusing.

*Error has been corrected.*

Figure 7B (also not labelled as b) Patient 23 results: At first the results look interesting but upon closer examination two other points are 0. The lines seem to trick us that there is some correlation.

*The reviewer is correct in pointing out that the correlation between CTC and clinical course may be artefactual. We have inserted the following text to alert the reader to this possibility (line 376-378):*

*“No CTCs were detected on the fifth sampling; however, since false negatives are common, we cannot rule out that the possibility that the apparent correlation with the imaging data is an artifact.”*
